# Multidisciplinary Approach in the Management of a Complex Case: Implant-Prosthetic Rehabilitation of a Periodontal Smoking Patient with Partial Edentulism, Malocclusion, and Aesthetic Diseases

**DOI:** 10.1155/2017/6348570

**Published:** 2017-03-21

**Authors:** Alessandro Lanza, Fabrizio Di Francesco, Gennaro De Marco, Fabio Scognamiglio, Valeria Aruta, Angelo Itro

**Affiliations:** ^1^Multidisciplinary Department of Medical, Surgical and Dental Sciences, Campania University Luigi Vanvitelli, Via Luigi De Crecchio 7, 80138 Naples, Italy; ^2^Dental Prosthesis and Implantology, Multidisciplinary Department of Medical, Surgical and Dental Sciences, Campania University Luigi Vanvitelli, Via Luigi De Crecchio 7, 80138 Naples, Italy; ^3^Department of Humanities, University of Naples Federico II, Via Porta di Massa 1, 80133 Naples, Italy

## Abstract

Complex periprosthetic cases are considered as challenges by clinicians. Clinical and radiographic parameters should be considered separately to make the right choice between an endodontically or periodontally compromised treated tooth and implant. Therefore, in order to decide whether the tooth is safe or not, data that have to be collected are specific parameters of both the patient and the clinician. In addition, the presence of periodontal, prosthetic, and orthodontic diseases requires patients to be set in multidisciplinary approach. The aim of this case report is to describe how the multidisciplinary approach could be the best way to manage difficult cases of implant-prosthetic rehabilitation. How to rehabilitate with fixed prosthesis on natural teeth and dental implants a smoker patient who presents with active periodontitis, multiple edentulous areas, dental malocclusion, and severe aesthetic problems was also described.

## 1. Introduction

Nowadays, both fully [1, 2] and partially edentulous patients [3] can benefit from implant-prosthetic rehabilitation. This discipline has been demonstrating an increase of predictability and success throughout the years, being a valid alternative to prosthetic rehabilitation on natural teeth. However, it has to still be decided whether the right choice is an endodontically or periodontally compromised treated tooth or implant. It can be stated that periodontally compromised patients can take advantage of prostheses on implants, provided that periodontitis has been treated and supervised [4]. Nevertheless, this group of patients can experience disadvantages because the risk of peri-implantitis seems to be higher [5]. The aim of this case report is to describe how the multidisciplinary approach could be the best way to manage difficult cases of implant-prosthetic rehabilitation. In addition, implant-prosthetic rehabilitation on natural teeth and dental implants of a smoker patient who presents with active periodontitis, multiple edentulous areas, dental malocclusion, and severe aesthetic problems was described.

## 2. Case Presentation 

**Figure 1:**
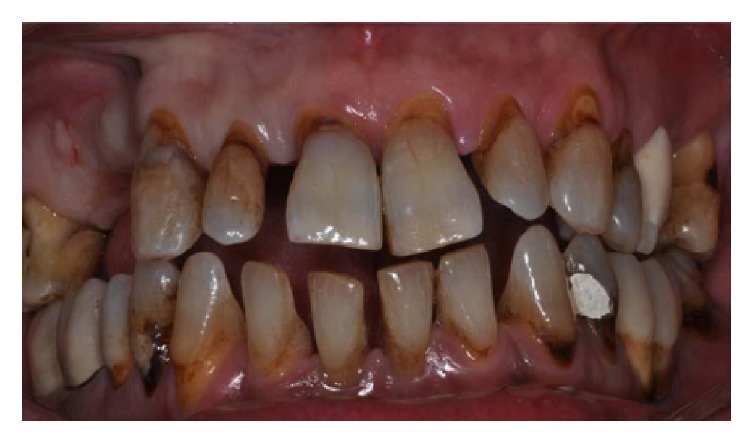
Starting case, clinical frontal view of the patient.

**Figure 2:**
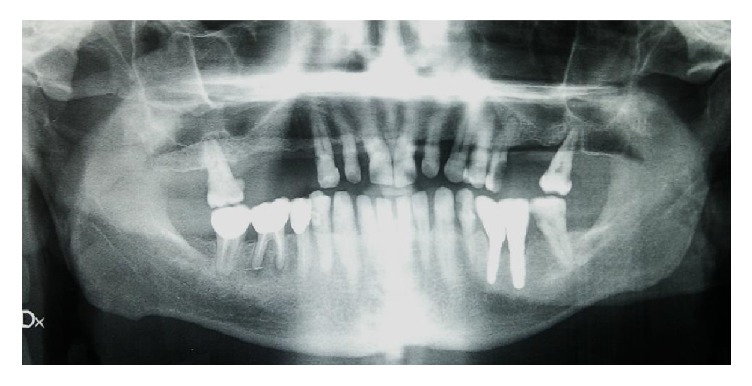
Starting case, full-mouth intraoral radiographic exam.

**Figure 3:**
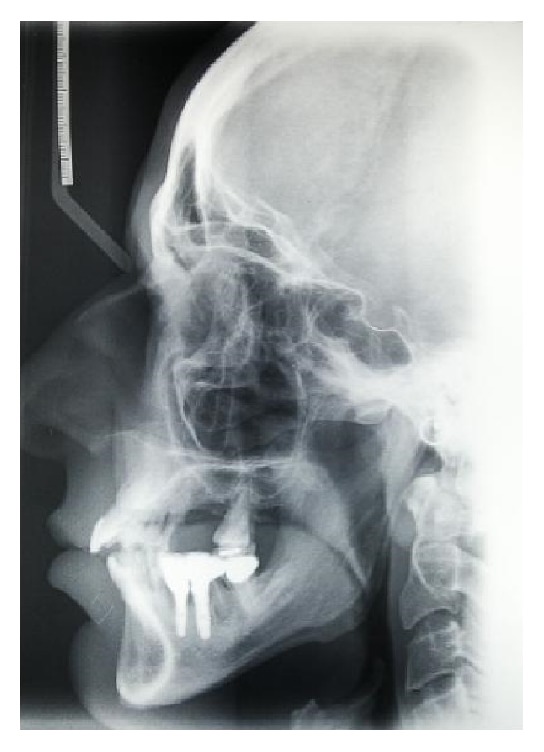
Starting case, teleradiographic exam.

**Figure 4:**
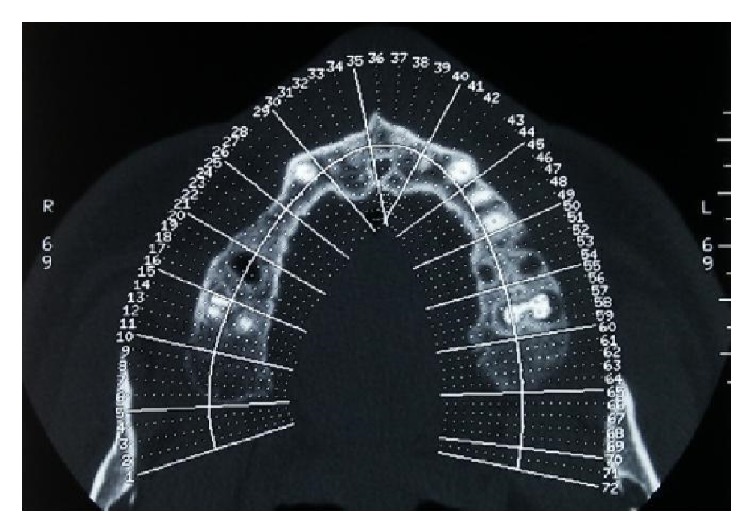
Initial case, TC Dentascan, axial view.

**Figure 5:**
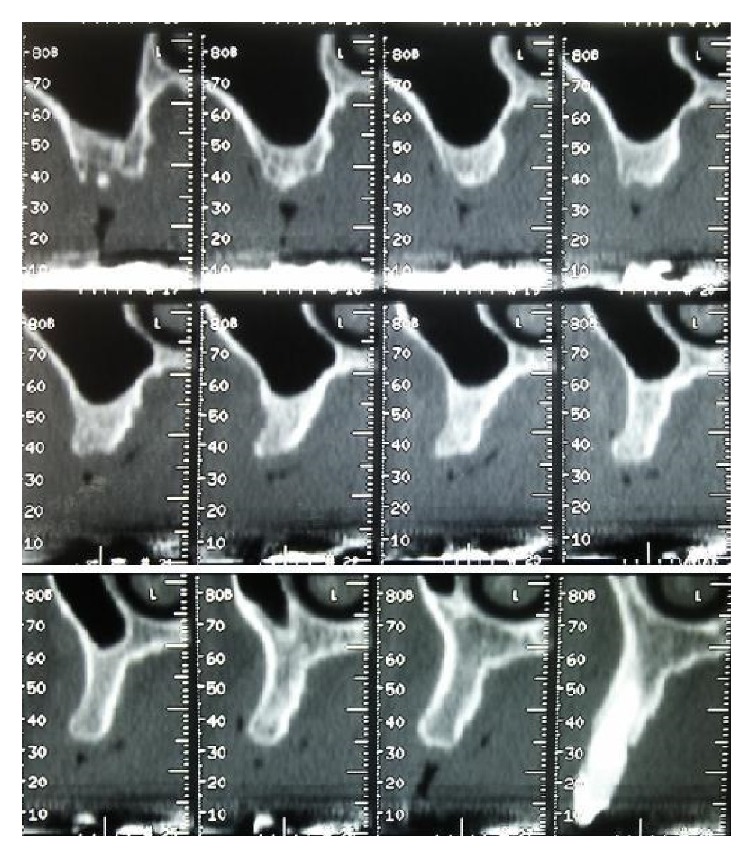
Initial case, TC Dentascan, cross-sectional images perpendicular to alveolar process of the maxilla.

**Figure 6:**
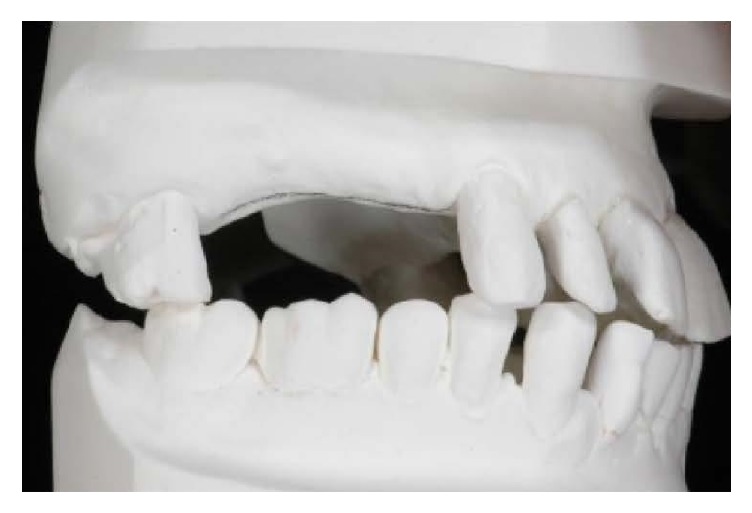
Analysis of edentulous space.

**Figure 7:**
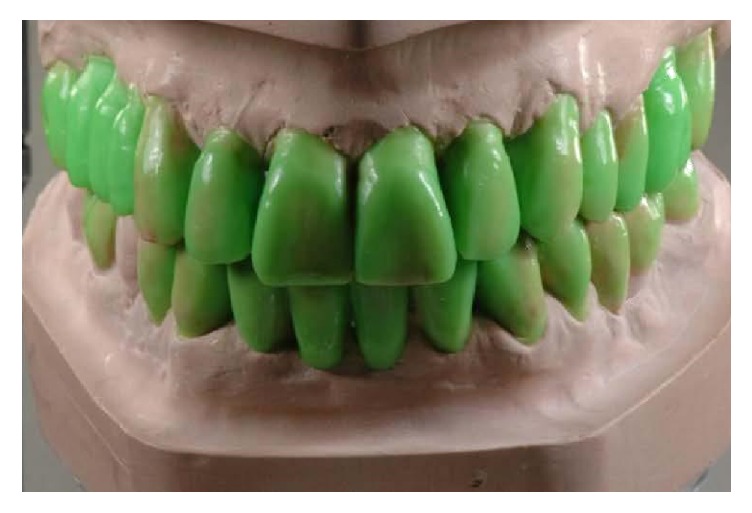
Diagnostic wax-up, frontal view.

**Figure 8:**
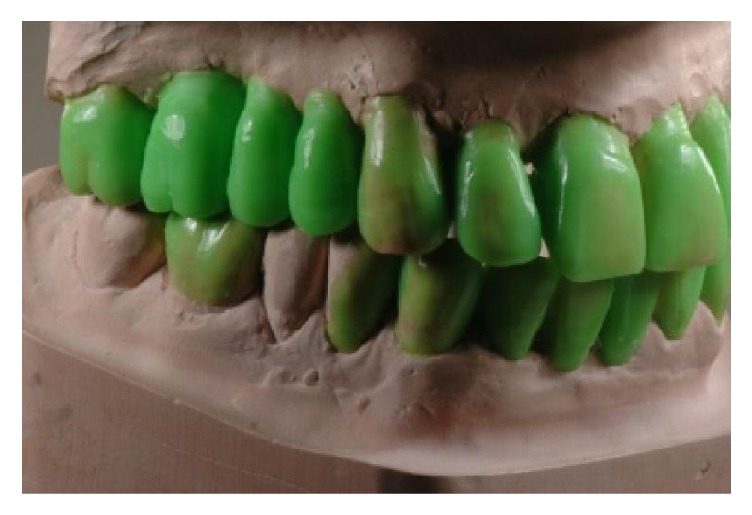
Diagnostic wax-up, lateral right view.

**Figure 9:**
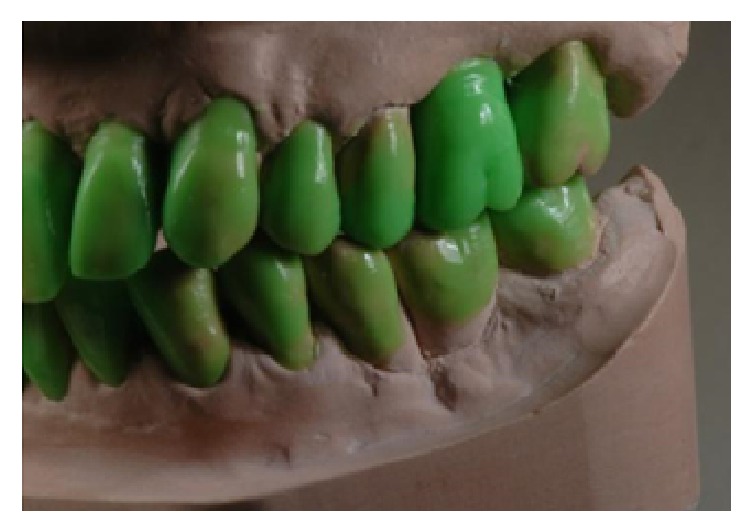
Diagnostic wax-up, lateral left view.

A 46-year-old male patient, smoker (more than 20 cigarettes per day), presented with numerous problems related to the bad condition of his oral cavity, in particular, pain and masticatory limitation, difficulty to pronounce certain words or letters, and inadequacy of the aesthetic appearance. The patient was in good health general state. Considering the clinical exam (Figure 1), radiographic exams (Figures 2, 3, 4, and 5), and examination of models (Figure 6), specialists in periodontics, implantology, prosthetics, and orthodontics made a diagnosis suggesting that the multidisciplinary approach was the best therapeutic choice to adopt for this complex case. A simulation of the final rehabilitation through a diagnostic wax was realized (Figures 7, 8, and 9) and analysed with the patient, aiming at highlighting what the advantages and disadvantages of the prostheses would be. Treatment objectives included the reestablishment of periodontal health through the elimination of etiological factors and the creation of a stable occlusal scheme for function; the programme included the development of fixed aesthetics rehabilitation on natural teeth and dental implants aimed at improving the aesthetic and functional aspect of the patient. Throughout the periodontal therapy, the patient was maintained periodontally stable and at each control he was motivated to oral hygiene, according to the guidelines set out recently in the seventh European Workshop on Periodontology [6].

**Figure 10:**
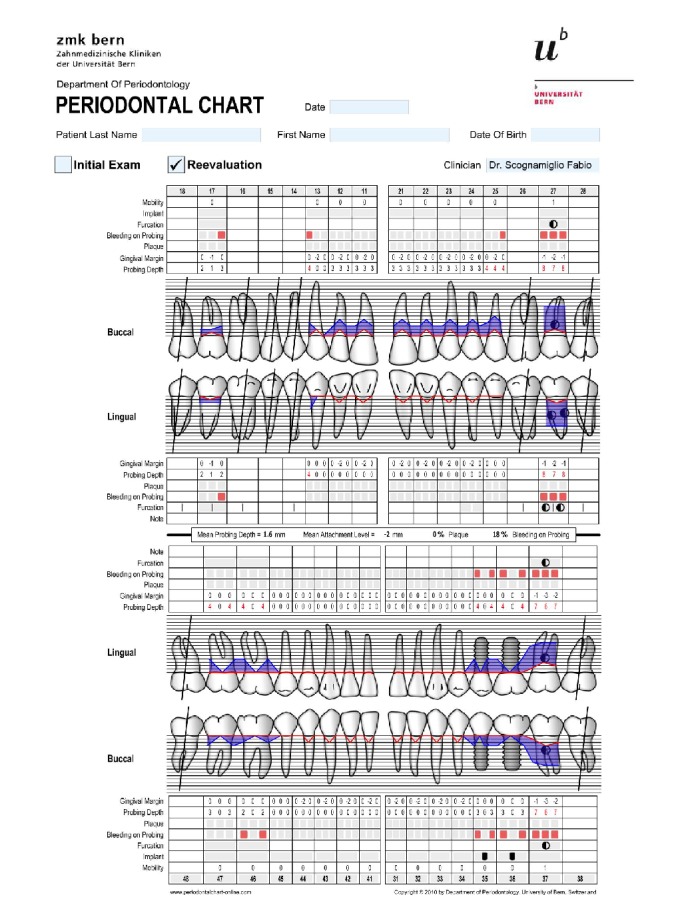
One month after nonsurgical periodontal therapy.

**Figure 11:**
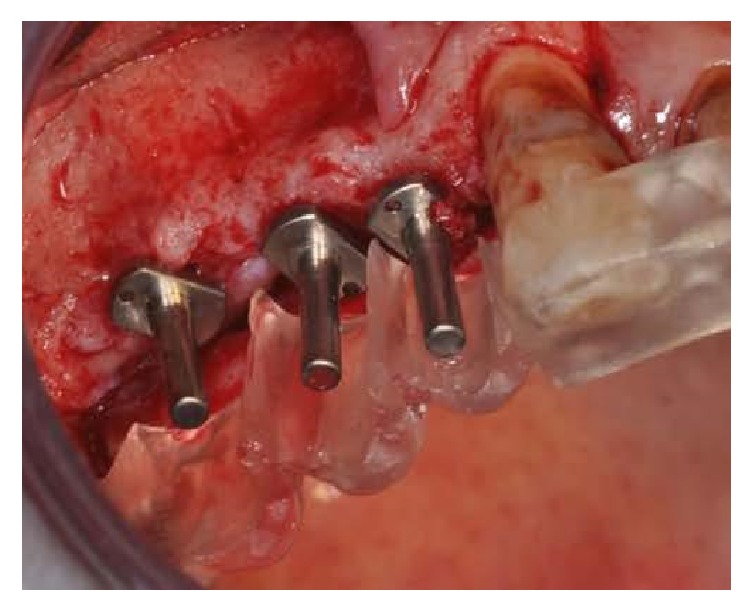
Inserted pins.

**Figure 12:**
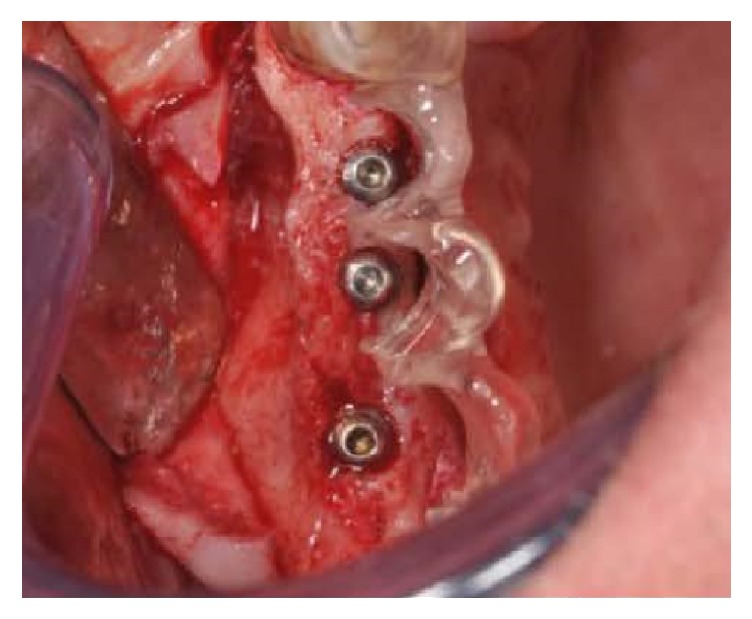
Implants placement.

**Figure 13:**
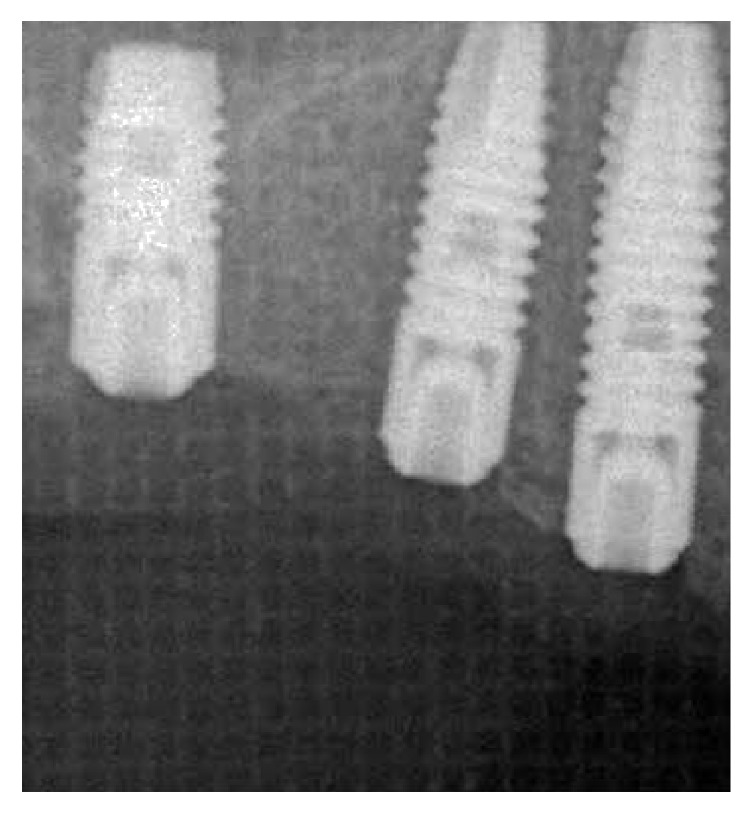
Rx control post-op.

**Figure 14:**
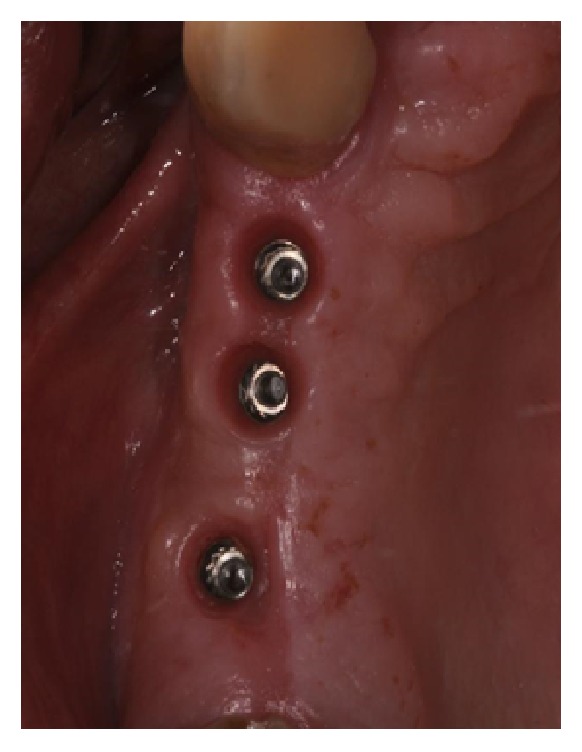
Conditioned soft tissue.

**Figure 15:**
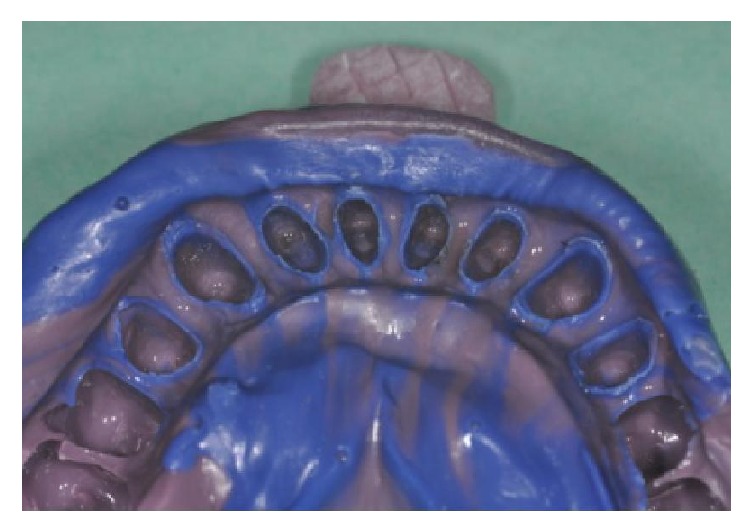
Lower final impression.

**Figure 16:**
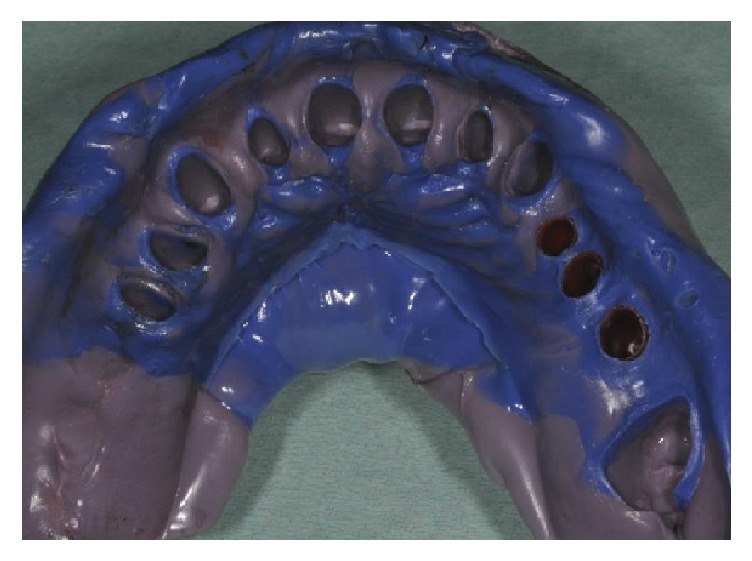
Superior final impression.

**Figure 17:**
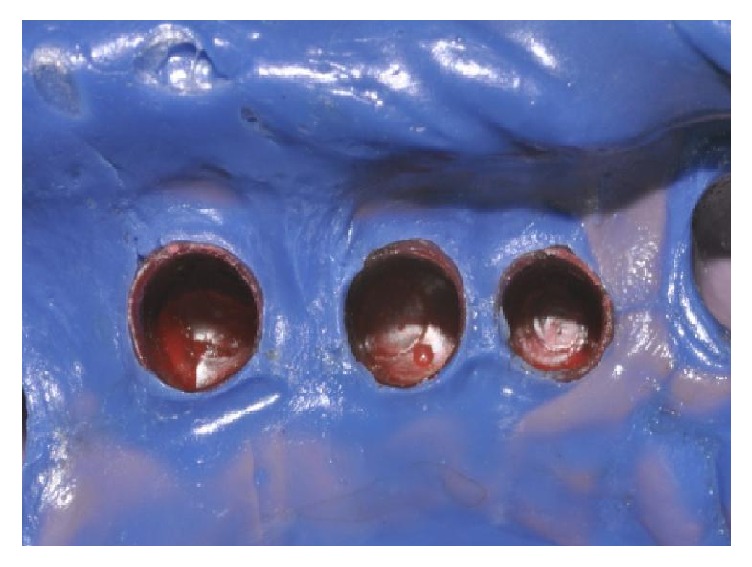
Details of superior final impression.

**Figure 18:**
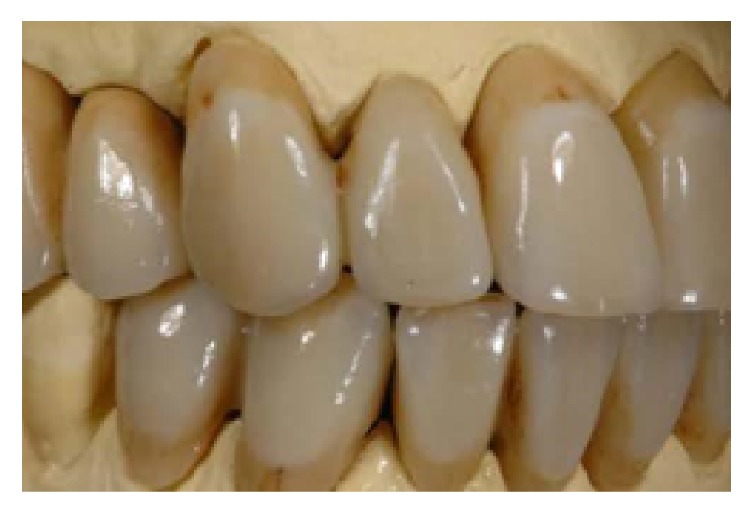
Final restorations, lateral right view.

**Figure 19:**
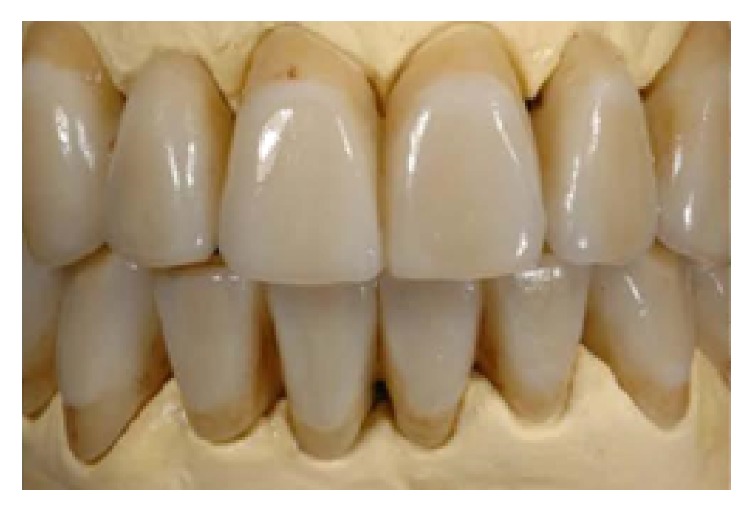
Final restorations, frontal view.

**Figure 20:**
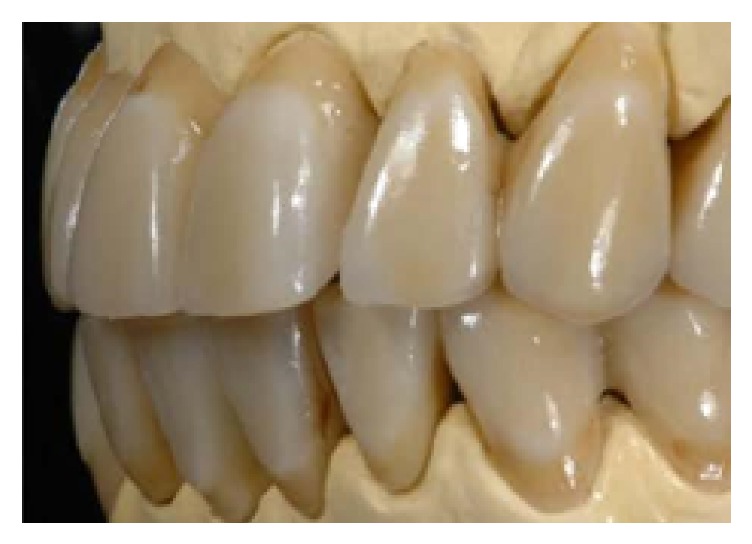
Final restorations, lateral left view.

One month after nonsurgical periodontal therapy had been completed (Figure 10), implant surgery was carried out. Implants insertion was completed in one surgical time under local anesthesia. Paracrestal full-thickness flaps were elevated; consequently the osteotomy was realized following the surgical stent (Figure 11). Three implants (Astra Tech OsseoSpeed TX Dentsply) were inserted in a prosthetic guided position [7] in site 1.4 with implant 3.5 mm in diameter and 13 mm in length, in site 1.5 with implant 3.5 mm in diameter and 11 mm in length, and in site 1.6 with implant 4.0 mm in diameter and 9 mm in length, respecting the interimplant distance of 2-3 millimeters (Figure 12) according to Tarnow et al. and Elian et al. [8, 9], throughout the implant length to influence in a positive way osseointegration (Figure 13). All implants achieved good primary stability with an insertion torque at least of 35 Ncm. During the healing phase, natural teeth were prepared and abutments were then covered with a provisional prosthesis to condition soft tissues, whereas 2.7 and 3.7 were extracted, regarded as hopeless. After four months necessary to osteointegration and conditioning soft tissue (Figure 14) the impression was taken for the definitive rehabilitation. Periodontal probing was within normal limits, and no inflammation was recorded neither around the teeth nor around implants (Figures 15, 16, and 17). Thus, a final impression was taken using a single-phase technique with double components polyether and individual tray. Lithium disilicate crowns were realized with the cross-mounting technique (Figures 18, 19, and 20): the occlusal scheme was designed with anterior guidance allowing complete disclusion in both the lateral and protrusive excursions. The final restoration included a tooth-borne fixed partial denture from teeth 1.3 to 2.5 and from teeth 4.4 to 3.4 and one implant-supported fixed partial denture from implant 1.6 to 1.4. The photographs were taken using Nikon D90 and a 105 mm lens (AF Micro Nikkor 105 mm 1 : 2.8 D, Nikon) with a ring flash (EM-140 DG, SIGMA-Nikon).

**Figure 21:**
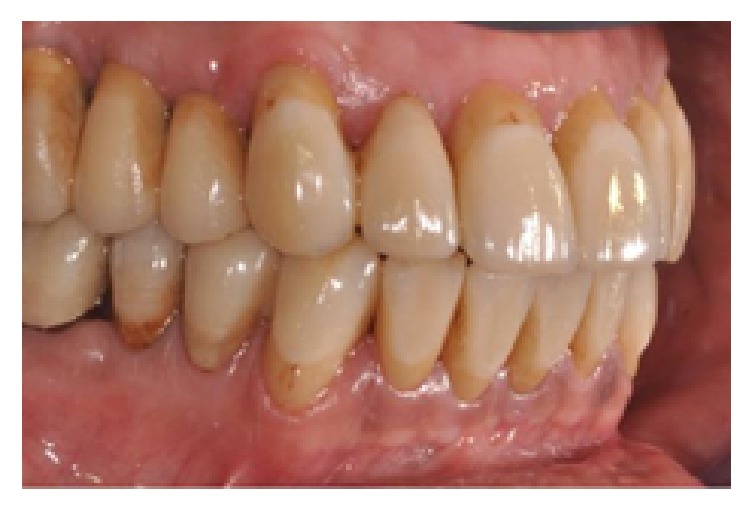
Lateral right view.

**Figure 22:**
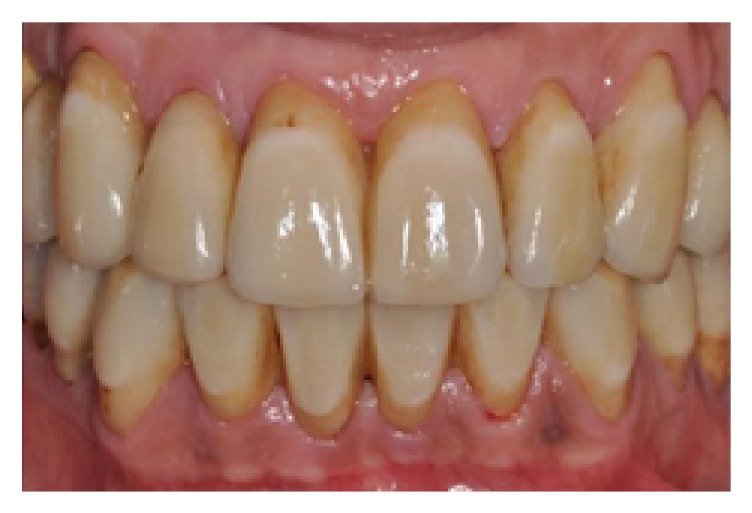
Frontal view.

**Figure 23:**
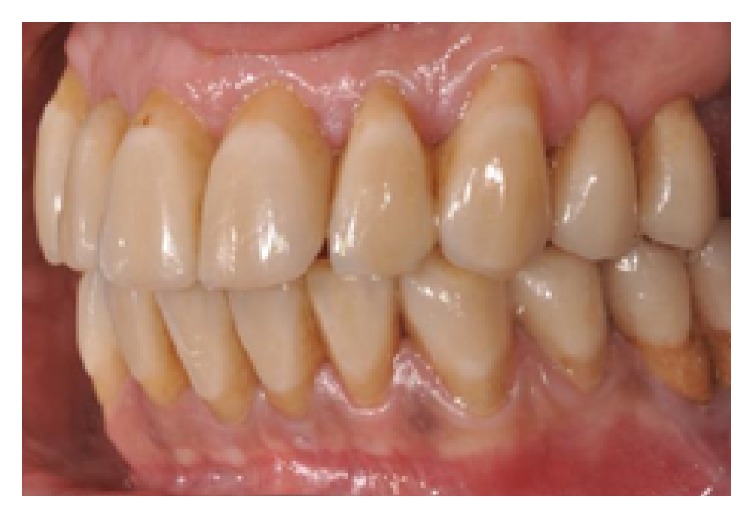
Lateral left view.

**Figure 24:**
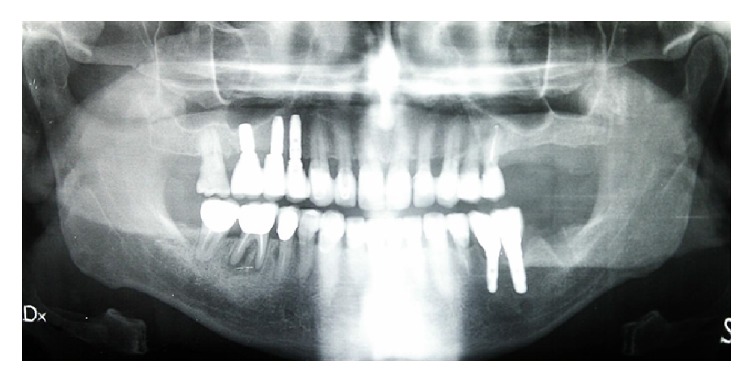
Final case, full-mouth intraoral radiographic exam.

At the end of the treatment, a maxillary retained night guard was provided to prevent any possible negative effect of parafunctional habits. According to Brägger et al. [10] the risk of incidence of complications increases in prosthetic rehabilitation due to parafunctions. From a functional and esthetic perspective, the objectives were achieved restoring good occlusal stability and a pleasant and harmonious smile line (Figures 21, 22, and 23). Periodic nonsurgical periodontal therapy and accuracy of prosthetic structures helped maintain the amount of bone fairly stable over time. The radiographic check at one year (Figure 24) demonstrated no bone loss around implants and teeth compared to the starting condition (Figure 2). The multidisciplinary approach [11] together with the control of local risk factors such as plaque and smoke [12] has allowed obtaining an aesthetic and functional integration of the final rehabilitation on natural teeth and implants.

## 3. Discussion 

Complex periprosthetic cases are considered as challenges by clinicians. A proper diagnostic setup is the starting point to develop an efficient treatment plan. Thus, the predictability of the therapy and its advantages and disadvantages support the clinician to assess the prognosis. It is extremely important to make the right choice between an endodontically or periodontally compromised treated tooth and implant. Therefore, in order to decide whether the tooth is safe or not, data that have to be collected are probing depth, attachment level, mobility, inflammatory and hygiene indices, root anatomy, furcation involvement, and crown-to-root ratio [13, 14], but not enough. Avila et al. [15] consider six levels of evaluation to decide whether a tooth can be saved or not; for each level a number of variables are assigned with the green, yellow, and red colours to indicate long-term maintenance favorable, caution recommended, and long-term survival unfavorable, respectively; among these variables as well as parameters related to the specific site, variables relating to systemic condition, compliance, and expectations of the patient together with clinician's skill can be noted. According to their scores, then a point total was assigned, expression of a clinical indication which may be recommended/considered extraction or maintenance of the treated tooth [15]. Evaluation of these parameters enables the clinician to determine the role that every single element can play in the prosthetic rehabilitation of the case [13–15]. According to above-mentioned studies, the decision to save a tooth or not is determined by specific parameters of both the patient and the clinicians. In the current case, molars in the second and third quadrant have a negative prognosis due to severe periodontal destruction, so the extraction of them was preferred reducing a chewing up to 2.5 to 3.6. On the other hand, it can be stated that periodontally compromised patients can benefit from prostheses on implants, provided that periodontitis has been treated and supervised, although the risk of peri-implantitis seems to be higher [4, 5]. Only through a starting periodontal therapy to eliminate etiological factors and regular follow-up to maintain the stability of periodontally conditions, the implant therapy could be considered predictable in periodontal patient. In addition, the presence of orthodontic problems and bad habits that could concern these patients requires them to be set in multidisciplinary approach.

## 4. Conclusions 

The multidisciplinary approach is the best way to manage difficult cases of implant-prosthetic rehabilitation. A proper diagnostic setup is the starting point to develop an efficient treatment plan; however, the predictability of the therapy depends on different factors relating to patient and clinicians. Therefore, periodontal and prosthetic control of the case and good patient compliance are the key factors in order to increase the predictability of the multidisciplinary approach in advanced case of implant-prosthetic rehabilitation.

## References

[1] Sadowsky S. J., Hansen P. W. (2014). Evidence-based criteria for differential treatment planning of implant restorations for the mandibular edentulous patient. *Journal of Prosthodontics*.

[2] Sadowsky S. J., Fitzpatrick B., Curtis D. A. (2015). Evidence-based criteria for differential treatment planning of implant restorations for the maxillary edentulous patient. *Journal of Prosthodontics*.

[3] Romeo E., Chiapasco M., Ghisolfi M., Vogel G. (2002). Long-term clinical effectiveness of oral implants in the treatment of partial edentulism: seven-year life table analysis of a prospective study with ITI® Dental Implants System used for single-tooth restorations. *Clinical Oral Implants Research*.

[4] Zangrando M. S., Damante C. A., Sant'ana A. C., De Rezende M. L. R., Greghi S. L., Chambrone L. (2015). Long-term evaluation of periodontal parameters and implant outcomes in periodontally compromised patients: a systematic review. *Journal of Periodontology*.

[5] Aguirre-Zorzano L. A., Estefanía-Fresco R., Telletxea O., Bravo M. (2015). Prevalence of peri-implant inflammatory disease in patients with a history of periodontal disease who receive supportive periodontal therapy. *Clinical Oral Implants Research*.

[6] Lang N. P., Berglundh T. (2011). Periimplant diseases: where are we now?—Consensus of the Seventh European Workshop on Periodontology. *Journal of Clinical Periodontology*.

[7] Garber D. A., Belser U. C. (1995). Restoration-driven implant placement with restoration-generated site development. *Compendium of Continuing Education in Dentistry*.

[8] Tarnow D. P., Cho S. C., Wallace S. S. (2000). The effect of inter-implant distance on the height of inter-implant bone crest. *Journal of Periodontology*.

[9] Elian N., Bloom M., Dard M., Cho S.-C., Trushkowsky R. D., Tarnow D. (2011). Effect of interimplant distance (2 and 3 mm) on the height of interimplant bone crest: a histomorphometric evaluation. *Journal of Periodontology*.

[10] Brägger U., Aeschlimann S., Bürgin W., Hämmerle C. H. F., Lang N. P. (2001). Biological and technical complications and failures with fixed partial dentures (FPD) on implants and teeth after four to five years of function. *Clinical Oral Implants Research*.

[11] Goyal M. K., Goyal S., Hegde V., Balkrishana D., Narayana A. I. (2013). Recreating an esthetically and functionally acceptable dentition: a multidisciplinary approach. *The International Journal of Periodontics & Restorative Dentistry*.

[12] Heitz-Mayfield L. J. A. (2008). Peri-implant diseases: diagnosis and risk indicators. *Journal of Clinical Periodontology*.

[13] Tonetti M. S., Steffen P., Muller-Campanile V., Suvan J., Lang N. P. (2000). Initial extractions and tooth loss during supportive care in a periodontal population seeking comprehensive care. *Journal of Clinical Periodontology*.

[14] Landi L., Piccinelli S., Raia R., Marinotti F., Manicone P. F. (2016). Perioprosthetic and implant-supported rehabilitation of complex cases: clinical management and timing strategy. *Case Reports in Dentistry*.

[15] Avila G., Galindo-Moreno P., Soehren S., Misch C. E., Morelli T., Wang H.-L. (2009). A novel decision-making process for tooth retention or extraction. *Journal of Periodontology*.

